# Recombinant *Treponema pallidum* Protein Tp0965 Activates Endothelial Cells and Increases the Permeability of Endothelial Cell Monolayer

**DOI:** 10.1371/journal.pone.0115134

**Published:** 2014-12-16

**Authors:** Rui-Li Zhang, Jing-Ping Zhang, Qian-Qiu Wang

**Affiliations:** 1 Department of Dermatology, Wuxi Second Affiliated Hospital of Nanjing Medical University, Wuxi, Jiangsu Province, China; 2 Institute of Dermatology, Chinese Academy of Medical Sciences & Peking Union Medical College, & National Center for STD Control, China Centers for Diseases Control and Prevention, Nanjing, Jiangsu Province, China; University of Illinois at Chicago, United States of America

## Abstract

The recombinant *Treponema pallidum* protein Tp0965 (rTp0965), one of the many proteins derived from the genome of *T. pallidum* subsp. *pallidum*, shows strong immunogenicity and immunoreactivity. In this study, we investigated the effects of rTp0965 on the endothelial barrier. Treatment of human umbilical vein endothelial cells (HUVECs) with rTp0965 resulted in increased levels of ICAM-1, E-selectin, and MCP-1 mRNA and protein expression. These increases contributed to the adhesion and chemataxis of monocytes (THP-1 cells) to HUVECs preincubated with rTp0965. In addition, rTp0965 induced reorganization of F-actin and decreased expression of claudin-1 in HUVECs. Interestingly, inhibition of the RhoA/ROCK signal pathway protected against rTp0965-induced higher endothelial permeability as well as transendothelial migration of monocytes. These data indicate that Tp0965 protein may play an important role in the immunopathogenesis of syphilis.

## Introduction

The endothelial barrier, constituted by the vascular endothelial cells lining the inner lumen of blood vessels and capillaries, plays a crucial role in the health and integrity of tissues by regulating the passage of molecules, liquids and immune cells [1], [2], [3]. Dysfunction and/or disruption of this barrier can lead to edema, inflammation, and associated pathologies [4], [5]. Many pathogens, from viruses to bacteria and parasites, can break down the endothelial barrier by damaging the vascular endothelium [6], [7], [8]. They can also trigger the opening of the intercellular junction, induce apoptosis of endothelial cells or activate the immune system, which in turn leads to the destruction of the endothelial barrier and subsequent edema [9], [10], [11], [12].

Previous studies suggested that bacterial infections altered the function of endothelial cells. *Neisseria meningitides* [13], as well as *Staphylococcus aureus* [14], adhere to endothelial cells, and induce the expression of endothelial adhesion molecules (ICAM-1, E-selectin, and VCAM-1), which contribute to leukocyte attachment. *Leptospira*, through adhesion to endothelial cells, may disrupt endothelial barrier function and promote dissemination of the bacteria [15]. The outer surface lipoprotein A (OspA) of *Borrelia burgdorferi*, as shown with *Neisseria meningitides*, increases the expression of endothelial adhesion molecules (ICAM-1, E-selectin, and VCAM-1) of HUVECs and subsequently adds to the transendothelial migration of neutrophils in vitro [16].

Syphilis is a chronic systemic, sexually transmitted disease caused by the bacterial spirochete *Treponema pallidum* subsp. *pallidum* (*T. pallidum*). The lesions of tertiary syphilis present as skin nodules, aortitis, aortic aneurism, gumma, and meningovasculitis [17], [18], [19]. Histopathology of these lesions reveals endothelial cell swelling, mural edema, perivascular and interstitial lymphohistocytic infiltrate, and thrombosis. Most of the clinical and histopathological manifestations appear to be the consequence of dysfunction and disruption of the endothelial barrier. Thus, studies on the mechanism of endothelial barrier regulation are important for insight into pathogenic mechanism of *T. pallidum* infections.

Previous investigations reported that *T. pallidum* was capable of penetrating intercellular junctions of endothelial cell monolayers and mouse abdominal wall tissue barriers [20], [21]. The bacteria can activate directly host vascular endothelium, up-regulate the expression of adhesion molecules, and promote the adherence of T-lymphocytes to human dermal microvascular endothelial cells. These studies suggested that invading activated endothelial cells play an important role in the dysfunction and disruption of the endothelial barrier in primary and secondary syphilis. However, during later syphilis, it is difficult to demonstrate *T. pallidum* in some lesions (such as gumma and spinal cord). An immunologic attack, including allergic, hypersensitivity, and other factors was proposed [22], [23]. We propose that some antigens, especially those exposed during *T. pallidum* killing, may play an important role in dysfunctions or disruption of endothelium barrier. Although several *T. pallidum* outer member proteins have been shown to regulate the expression of cell adhesion molecules and binding of T-Lymphocytes to human dermal microvascular endothelial cells (HDMECs) [24], [25], there is little evidence for the role of other member proteins in dysfunctions or disruption of the endothelium barrier.

*T. pallidum* is an obligate human pathogen and cannot be cultivated in vitro. This has severely impeded progress in understanding precise pathogenesis of this microbe. The availability of the *T. pallidum* genome sequence made it possible to examine predicted *T. pallidum* open reading frames (ORFs) for potential suitability as diagnostic or immunization tools [26]. This approach permits identification of low-abundant *T. pallidum* antigens, since they may be expressed as recombinant proteins in much larger quantities. Several proteins have been characterized in involving in adhesion, invasion, and/or dissemination [27], [28], [29], [30].

Genomic analysis of *T. pallidum* suggestes that the *tp0965* gene is located on the *tp34* gene cluster (the *tp0959* to *tp0972* genes) [31]. Because of similar function, *T. pallidum* cluster the *tp34* gene cluster into *tp34* operon. The Tp0965 protein is encoded by *tp0965*, containing 320 amino acids, with a molecular weight of 35.4 kDa. The Protein BLAST data revealed that Tp0965 is a membrane fusion protein and is located on periplasm of *T. pallidum*. Previous studies suggest that the Tp0965 protein, together with other proteins encoded by the Tp34 operon, may compose a periplasma-spanning export system to provide transporters with various specificities [33]. Tp0965 is reactive with sera from syphilitic individuals at all stages and shows strong immunoreactivity. In our previous research, we gave rabbits subcutaneous injections of recombinant Tp0965 protein (rTp0965) and observed strong immunogenicity with the protein [32]. However, reports concerning the role of Tp0965 in the pathogenesis of syphilis are lacking.

We hypothesize that similar to that of other pathogens, the Tp0965 protein has effects on the function of the endothelial barrier and, to an extent, plays a role in the immunopathogenesis of late-stage syphilis. In the present study, we examine the effects of rTp0965 on expression and mRNA levels of adhesion molecules in HUVECs. In addition, we examine the changes in permeability of HUVEC monolayers and transendothelial migration of monocytes. We also see effects of rTp0965 on the reorganization of F-actin and expression of claudin-1. The results indicate that rTp0965 has the capability of triggering endothelial cell activation and regulates the function of the endothelial barrier.

## Materials and Methods

### Materials

Transwell tissue culture inserts (diameter, 6.5 mm; pore size, 5.0 µm) and 24-well plates were purchased from Corning Costar Corp (Corning, NY). RPMI 1640 medium and endothelial growth medium were obtained from Gibco (Grand Island, NY). Heat-inactivated fetal bovine serum was obtained from HyClone (Logan, Utah). Mouse anti-human claudin-1 was obtained from Cell Signaling Technology (America, Boston). The *Limulus* amebocyte lysate test kit, polymyxin B-agarose, anti-human ICAM-1 monoclonal antibody (MAb), anti-human E-selectin (MAb), Calcein AM, phalloidin, tetramethylrhodamine bisothiocyanate, matrigel matrix, and MCP-1 were purchased from Sigma-Aldrich (America, St Louis, MO). Recombinant *T. pallidum* protein Tp0965(rTp0965)was purified on nickel-nitrilotriacetic acid (Ni-NTA) chromatographic column from *E. coli* lysates frozen in our laboratory. The human MCP-1 enzyme-linked immunosorbent assay (ELISA) kit was purchased from R&D Systems, Inc. (Minneapolis, MN). Anti-mouse immunoglobulin G and horseradish peroxidase (HRP)-tagged antibody were purchased from Amersham (Piscataway, NJ). Anti-rabbit HRP-tagged antibody was purchased from Zymed (San Francisco, CA).

### Recombinant protein preparation

Recombinant protein Tp0965 was expressed in *E. coli* and purified as described previously [32]. Briefly, the gene of Tp0965 was amplified by polymerase chain reaction (PCR) from *T. pallidum* genomic DNA and the nucleotide sequence was cloned into the expression plasmid pET28a (Invitrogen, USA). The new constructs were transformed into *E. coli* Rosetta (DE3) (Stratagene, La Jolla, USA) and the recombinant fusion proteins were purified on Ni-NTA chromatographic column. The collected proteins were renatured by dialysis based upon the renaturation protocol described previously [34]. Sodium dodecyl sulfonate and polyacrylate gel electrophoresis (SDS-PAGE) and immunoblot analysis using the anti-polyhistidine tag antibody were employed to identify the protein and assess its purity. Protein concentrations were determined using a bicinchoninic acid (BCA) Protein Assay Kit (Sangon Biotech Co. Ltd., China). To remove LPS contamination, the recombinant protein was subsequently treated by polymyxin B-agarose and the LPS level was detected by the *Limulus* amebocyte lysate test kit.

### Cell culture

Human umbilical vein endothelial cells (HUVECs) were a kind gift from Dr. HL Li (China Third Military Medical University, ATCC, PCS-100-100), and routinely cultured as specified by the Dr. Li in endothelial growth medium (EGM) containing 0.2% bovine brain extract, 5 ng/ml human EGF, 10 mM L-glutamine, 1 µg/ml hydrocortisone, 2% FBS, and 0.5% penicillin/streptomycin. Monocyte THP-1 cells were frozen in our laboratory and were routinely cultured in RPMI 1640 medium supplemented with 10% FBS and 0.05 mM 2-mercaptoethanol.

### ELISA

HUVECs were seeded on 96-well plates at a concentration of 5×10^4^ cells per well and incubated at 37°C in 5% CO_2_ for 24 h. They were then treated with either rTp0965 or boiled rTp0965 (served as negative control) at 37°C in 5% CO_2_. The supernatants were collected for determining the level of MCP-1 by ELISA according to the manufacturer's instructions (BD Biosciences). Final results were read at a wavelength of 450 nm.

### Real-time reverse transcription-PCR (RT-PCR)

HUVECs were seeded on 96-well plates at a concentration of 5×10^4^ cells per well and incubated at 37°C in 5% CO_2_ for 24 h. They were then treated with either rTp0965 or boiled rTp0965 at 37°C in 5% CO_2_. The cells were harvested for mRNA transcripts of ICAM-1, E-selectin and MCP-1 with real-time RT-PCR. Briefly, total RNA was extracted with TRIzol reagent (Invitrogen), and the RNA samples were treated with DNase I before reverse transcription processing to remove genomic DNA contamination. A total of 2 µg RNA from each sample was reverse transcribed into cDNA with the AMV First Strand cDNA Synthesis Kit (BBI) according to the manufacturer's protocol. The levels of mRNA transcripts were analyzed on an ABI Stepone Plus Sequence Detection System (Applied Biosystems) following a one-step quantitative reverse transcriptase–polymerase chain reaction (qRT-PCR) using specific primers, along with the SYBR Green PCR Master Mix and RT-PCR kit according to the manufacturer's instructions. Glyceraldehyde-3-phosphate dehydrogenase (GAPDH), a housekeeping gene, was used as the internal control. Reaction volumes of 20 µl containing 50 ng of total RNA, 10 µl 2x SYBR Green PCR Master Mix, and 10 µM primers were subjected to one cycle of 95°C for 5 min and then 40 cycles of 95°C for 10 s, 60°C for 30 s and 72°C for 45 s. For relative quantification, the levels of individual mRNA transcripts were first normalized to the level of the control GAPDH mRNA. The differential expression of these genes was then analyzed by the ΔCt method and expressed as fold change. The amplified products were analyzed by 2% agarose gel electrophoresis.

### Cell ELISA

HUVECs (2×10^4^ cells/well) were seeded into 96-well plates and cultured for 24 h. Then, HUVECs were cultured with either rTp0965 or boiled rTp0965. After incubation, HUVECs were washed with PBS and were fixed with paraformaldehyde 4% in PBS. Then HUVECs were blocked with bovine serum albumin (BSA) 1% for 2 h. The cells were incubated for 2 h with anti-human ICAM-1 MAb or anti-human E-selectin MAb at a dilution of 1 in 200. The thrice-washed cells were incubated for 2 h with biotin-conjugated goat anti-rabbit IgG at a dilution of 1 in 50 and incubated with avidin-conjugated horseradish peroxidase for 30 min. The assay was developed by addition of TMB peroxidase EIA substrate. Final results were read at a wavelength of 450 nm.

### Adherence assay

HUVECs were grown to confluence over 24 h on 24-well plates, preincubated with rTp0965 (800 ng/ml) or boiled rTp0965 (800 ng/ml) at 37°C in 5% CO_2_ for 24 h. Monocyte THP-1 cells were stained with calcein AM (5 µM, for 25 min) and added in the amount of 5×10^5^ cells to each well and incubated for a further 6 h at 37°C in 5% CO_2_. After washing with PBS, the monocyte THP-1 cells in either supernatant or PBS were counted under the fluorescence microscope. Monocyte THP-1 cells adhering to endothelial cells were calculated as follows: percentage of monocyte THP-1 cells binding  =  (counts of cells added per well – counts of cells uncombined)/(counts of cells added per well) ×100.

### Chemotaxis assay

The chemotaxis assay was performed in 24-well Transwell inserts (6.5 mm and 5.0 µm). Briefly, HUVECs were seeded on the wells to confluence over 24 h, and treated by rTp0965 (800 ng/ml) or boiled rTp0965 (800 ng/ml), and then incubated for 24 h at 37°C in 5% CO_2_. Monocyte THP-1 cells were stained with calcein AM (5 µM, for 25 min) and added in the amount of 5×10^5^ cells in the inserts for 2 h at 37°C in 5% CO_2_, and the number of monocyte THP-1 cells in the wells were counted with a fluorescence microscope.

### Western blot

HUVECs were seeded into 96-well plates at a concentration of 5×10^4^ cells per well and incubated at 37°C in 5% CO_2_ for 24 h. They were treated with either rTp0965 (800 ng/ml) or boiled rTp0965 (800 ng/ml) for 24 h at 37°C in 5% CO_2_. The cells were harvested for detection of claudin-1 expression by Western blot. In some experiments, HUVECs were pre-incubated with the ROCK inhibitor Y-27632 (10 µM) for 30 mins at 37°C before HUVECs were harvested and Western blot was performed. Briefly, the harvested HUVECs were extracted with cold RIPA buffer. Equal amounts of proteins of the cell lysates were fractionated on 8% SDS–polyacrylamide gels (SDS–PAGE), electrophoretically transferred to nitrocellulose membranes, and subjected to Western blot analysis. After blocking in 2% BSA for 1 h, the membranes were incubated with primary antibodies for claudin-1 (1∶1000) as a loading control overnight at 4°C with gentle rocking and then incubated with HRP-conjugated anti-mouse antibodies at room temperature for 1 h. The bound antibodies were visualized under the image analysis software, and the level of each protein relative to β-actin was determined.

### Fluorescent staining

To analyze F-actin distribution, HUVECs were cultured on collagen-coated coverslips until confluent, then rTp0965 (800 ng/ml) or boiled rTp0965 (800 ng/ml) were added and the cells incubated for 24 h at 37°C. At the end of this incubation period, HUVECs were fixed in 4% buffered paraformaldehyde and permeabilized with 0.1% Triton X-100 in PBS containing 1% BSA, then stained for 2 h at room temperature with rhodamine-phalloidin (10 U/ml) in PBS. After washing with PBS, several drops of 90% glycerol/10% PBS were added. Finally, the coverslips were examined using a confocal laser scanning microscope system (Olympus FV1000, Japan). In some experiments, HUVECs monolayers were preincubated with the ROCK inhibitor Y-27632 (10 µM) for 30 mins at 37°C before F-actin distribution was tested as described above.

### HUVECs monolayer permeability and transendothelial migration assays

For endothelial permeability and transendothelial migration measurements, HUVECs were cultured on 6.5 mm and 5.0 µm Transwell inserts that have been coated with matrigel matrix for 7-10 days until confluent. Transendothelial electrical resistance (TEER) of HUVECs monolayers was measured and the inserts with stable values of TEER were chosen for the following assays. The cells were treated with rTp0965 (800 ng/mL) or boiled rTp0965 for 24 h, then the inserts were placed in the wells containing serum-free EGM.

For endothelial permeability, HRP (500 ng/mL) was added on top of the HUVEC monolayers and 50 µl samples were taken from the wells at 0.5 h, 1 h, 2 h, 3 h, and 4 h, respectively. Then 50 µl of fresh EGM was added to each well. The collected samples were analyzed for the flux of HRP with a TMB kit performed according to the manufacture's instructions (Sangon Biotech).

For the transmigration assay, treated cells growing on 5.0 µm membrane inserts were placed in the wells containing serum-free endothelial basal medium with CCL-2 (100 ng/mL) added as a chemoattractant. Monocyte THP-1 cells were stained with calcein AM (5 µM, for 25 min) and added in the amount of 5×10^5^ cells on top of HUVEC monolayers for 6 h. Next, the numbers of monocyte THP-1 cells in the wells and beneath the HUVEC monolayers were counted using a fluorescence microscope. In some experiments, HUVEC monolayers were princubated with the ROCK inhibitor Y-27632 (10 µM) for 30 mins at 37°C before the endothelial permeability and transendothelial migration assays were performed as described above.

### Statistics

Results were expressed as the mean ± standard deviations (SD) of experimental groups. For comparison of the mean values between two groups, the unpaired *t*-test was used. To compare values among multiple groups, one-way analysis of variance (ANOVA) was applied. *P*<0.05 was used as the alpha value to determine statistical significance for all analyses.

## Results

### Expression and purification of recombinant protein

**Figure 1:**
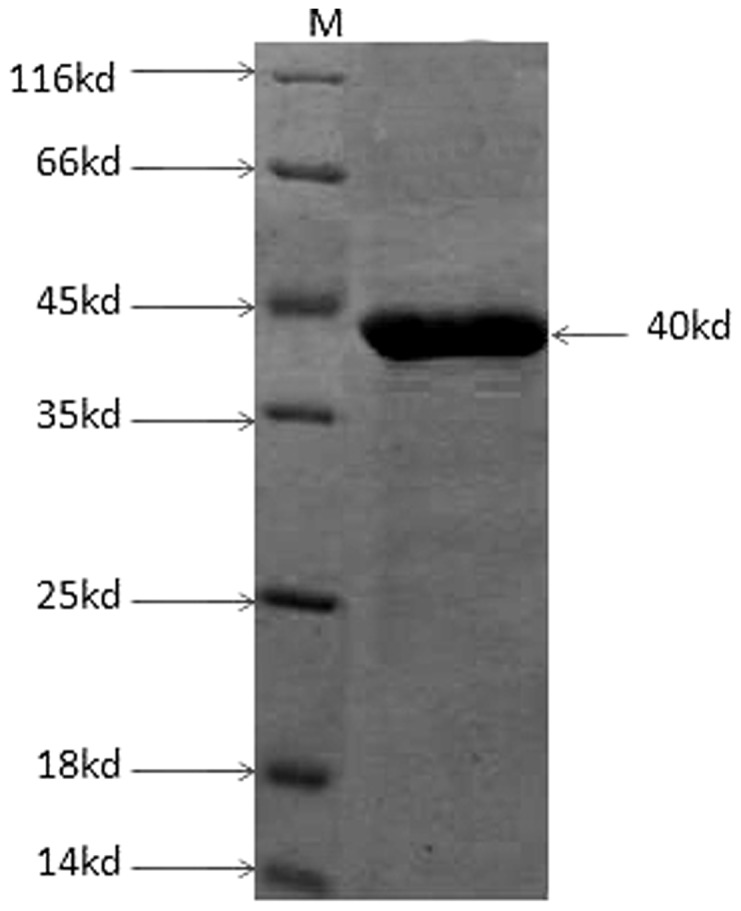
Recombinant Tp0965 protein, purified using Ni-NTA resin, analysed by 12% sodium dodecyl sulphate–polyacrylamide gel electrophoresis(SDS-PAGE). The electrophoresis showed a 40 kDa protein band. M: protein markers (lanes concentrations: 0.1∼0.2 mg/ml). Arrow indates the recombinant Tp0965 protein.

The *Tp0965* gene was successfully amplified from the *T. pallidum* Nichols strain genome and then cloned into the expression vector pET28a. Tp0965 was expressed in *E. coli* Rosetta (DE3) and purified from cell-free supernatants on Ni-NTA chromatographic column. The soluble protein Tp0965 was produced as a single protein that was determined by SDS-PAGE analysis to have an estimated purity of >90% (Fig. 1). The molecular mass in SDS-PAGE gel was estimated to be 40 kDa, a value that is significantly higher than the value expected from the deduced amino acid sequence. Increasing evidences showed that very basic or acidic proteins may migrate anomalously on SDS-PAGE gels [33]. Therefore, the anomalous migration of TP0965 might be due to abnormal binding of SDS to basic amino acids, since TP0965 is a Lys-rich basic protein, which in turn might lead to an abnormal shape of the SDS protein complex.

The identification of recombinant protein was performed by a Western blot assay with anti-polyhistidine tag antibody, human syphilis sera. These results showed that recombinant protein Tp0965 reacted positively with anti-His monoclonal antibody and human syphilis. The protein concentration was 1.1 mg/mL determined using a BCA Protein Assay Kit. After treated by polymyxin B-agarose, the final LPS level was lower than 2.0 EU/mL, which is an amount that did not stimulate proinflammatory cytokine production by itself.

### Effects of rTp0965 on adherence of monocytes to HUVECs

**Figure 2:**
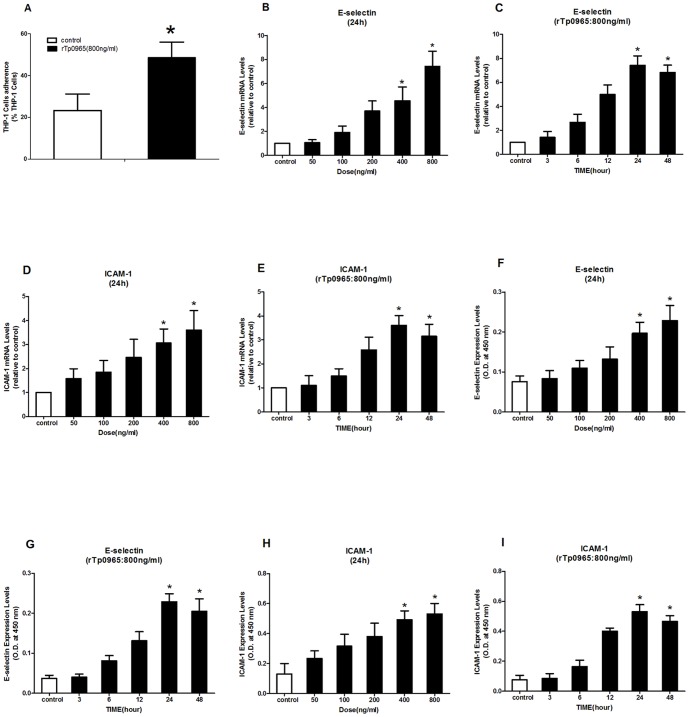
Effect of rTp0965 on adherence of monocytes to HUVECs. (A) Confluent HUVECs were stimulated with rTp0965 (800 ng/ml) for 24 h. Monocyte THP-1 cells stained with calcein AM were then added and incubated for 6 h. The percentage of THP-1 cells were counted under the fluorescence microscope. (B–E) Confluent HUVECs were incubated with various concentrations of the rTp0965 for increasing time intervals. The levels of ICAM-1 and E-selectin mRNA in HUVECs were assayed by RT-PCR. (F-I) Confluent HUVECs were incubated with various concentrations of rTp0965 for increasing time intervals. The expression levels of ICAM-1 and E-selectin were measured by cell ELISA. (* = *P*<0.05, compared to a control). Results shown are those of one experiment representative of three similar experiments.

LPS-enhanced retention and adhesiveness of human monocytes and monocytic cell lines to endothelium is seen in the immunopathogenesis of many infectious diseases. To test the effects of rTp0965 on monocyte adhesion to HUVECs, we pretreated confluent monolayers of HUVECs and then stimulated them with rTp0965 (800 ng/ml) for 24 h, followed by incubation with THP-1 cells for 1 h at 37°C. As shown in Fig. 2A, rTp0965 stimulated an increase in adherence of THP-1 cells to HUVECs (48.5±7.5% versus 23.3±7.9%, *P*<0.05). To analyze the expression of adhesion molecules on the HUVEC response to rTp0965, cell ELISA and real-time RT-PCR were used. As shown in Fig. 2B–I, real-time RT-PCR study showed that the mRNA transcription levels of ICAM-1 and E-selectin were increased significantly after incubation with rTp0965 (800 ng/ml) ( *P*<0.05) compared to controls. The up-regulation of transcription was increased in a dose-dependent manner by the various concentrations of rTp0965. Time course experiments identified the duration of incubation lead to maximal expression of adhesion molecules on HUVECs. The transcription of ICAM-1 and E-selectin peaked at 24 h. Cell ELISA analysis showed similar results. The rTp0965 induced a remarkable increase of ICAM-1 and E-selectin, compared to controls. These data indicated that rTp0965 could significantly induce up-regulation of adhesion molecules on HUVECs, and it also promoted an increased adherence of monocyte THP-1 cells to HUVECs.

### Effects of rTp0965 on chemoattraction of monocytes to HUVECs

**Figure 3:**
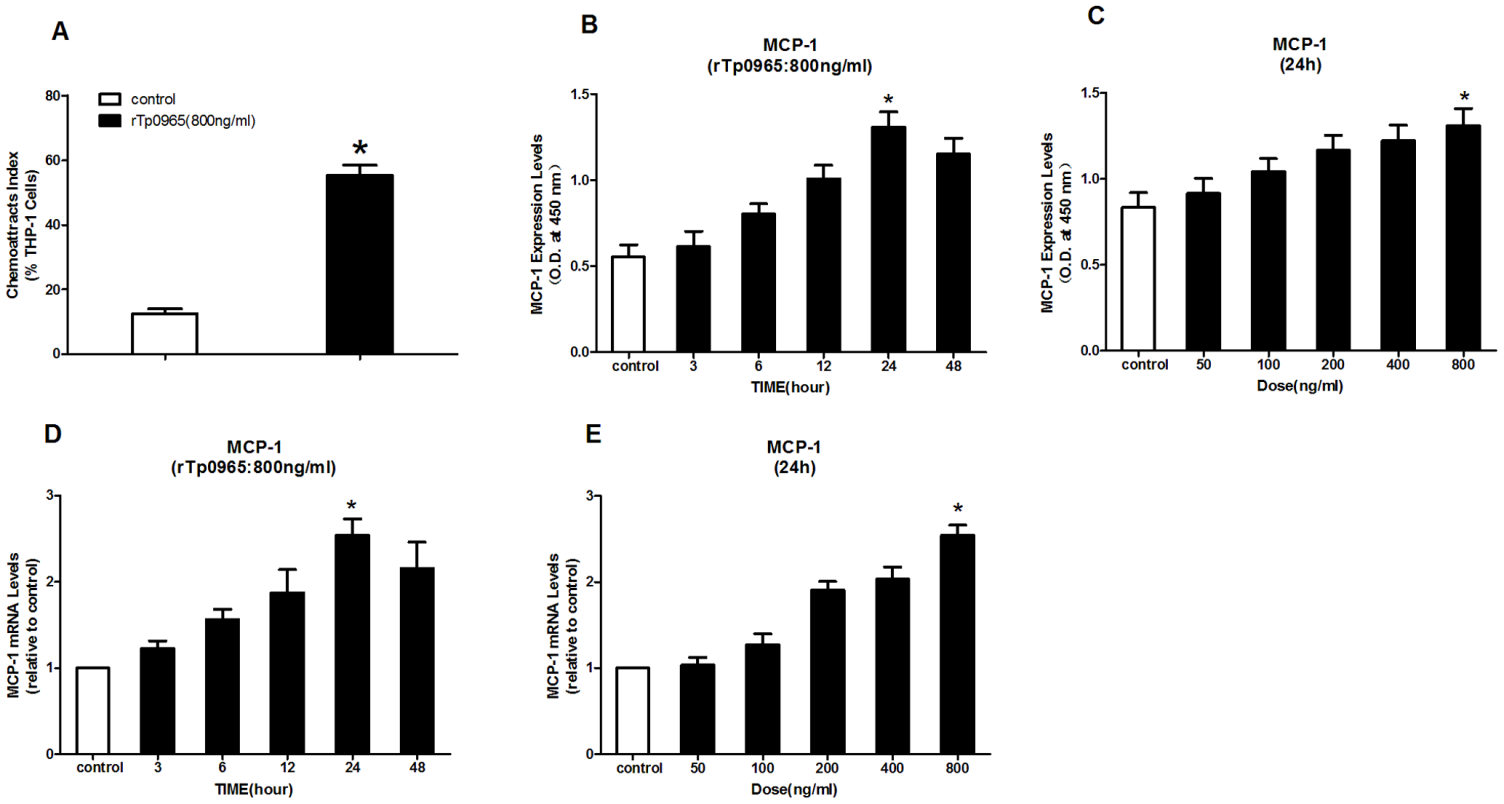
Effects of rTp0965 on chemoattraction of monocytes to HUVECs. (A) Confluent HUVECs in the wells were preincubated with rTp0965 (800 ng/ml) for 24 h. Monocyte THP-1 cells stained with calcein AM were then added in the inserts for 2 h and the percentage of THP-1 cells in the wells were counted under the fluorescence microscope. (B and C) Confluent HUVECs were incubated with various concentrations of the rTp0965 for increasing time intervals. The levels of MCP-1 in the supernatants were determined by ELISA. (D and E) Confluent HUVECs were incubated with various concentrations of rTp0965 for increasing time intervals. The levels of MCP-1 mRNA in HUVECs were measured by RT-PCR. (* = *P*<0.05, compared to a control). Results shown are those of one experiment representative of three similar experiments.

To examine the effects of rTp0965 on HUVECs chemoattraction of monocytes, monocyte THP-1 cells were added to the inserts of transwell systems that contained HUVECs (that had been pretreated with rTp0965) in the wells. Monocyte THP-1 cells migration was monitored for 2 h. Some monocyte THP-1 cells migration was evoked by HUVECs in control group, but significantly more monocyte THP-1 cells migrated towards the HUVECs pretreated with rTp0965 (Fig. 3A). To study the effects of MCP-1 secreted by HUVECs on monocyte THP-1 cell migration to the HUVECs, the expression and mRNA transcription levels of MCP-1 were examined with ELISA and real-time RT-PCR. ELISA data showed that the amount of soluble MCP-1 was increased significantly after incubation with rTp0965 (800 ng/ml) compared with the control (*P<*0.05). The up-regulation of expression was increased in a concentration-dependent manner by rTp0965. Time course experiments identified the duration of incubation that achieved maximal expression of MCP-1 on HUVECs. The expression of MCP-1 was peaked at 24 h. Real-time RT-PCR analysis showed similar results. A remarkable increase of MCP-1 mRNA, compared to the control, was induced by rTp0965. Together with the data displayed in Fig. 3B-E, these results showed rTp0965 could significantly induce up-regulation of MCP-1 on HUVECs, and also increased HUVECs chemoattraction of monocyte THP-1 cells.

### Effects of rTp0965 on the permeability of HUVECs monolayers

**Figure 4:**
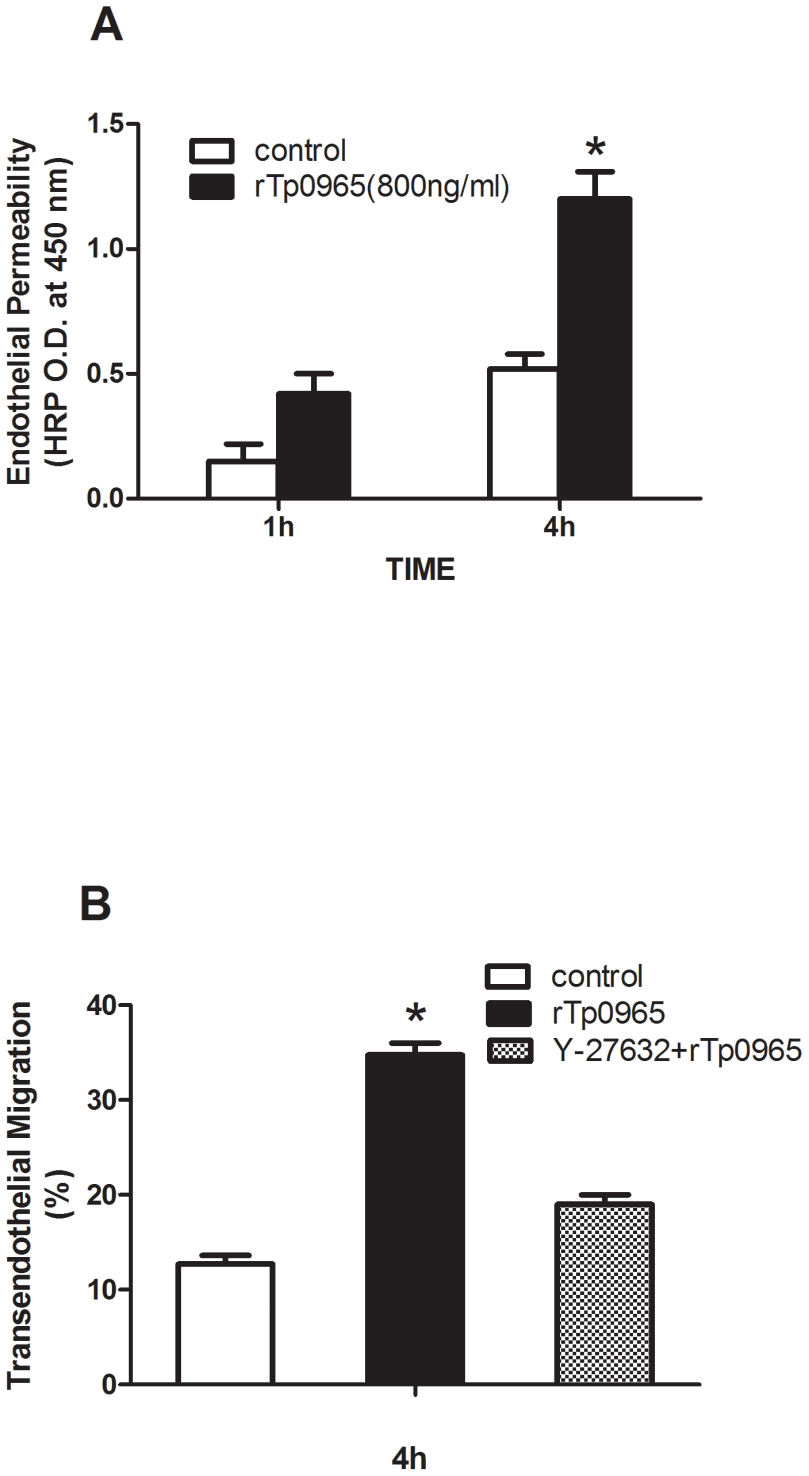
Effects of rTp0965 on the permeability of HUVEC monolayers. Confluent HUVECs in the wells were preincubated with rTp0965 (800 ng/ml) for 24 h. (**A**). HRP was then added to the transwell inserts and the flux of HRP in supernatants was calculated at 1 and 4h. (**B**) Effects of rTp0965 on migration of monocytes across HUVECs. Confluent monolayers of HUVECs cultured in Transwell inserts were pretreated with Y-27632 for 30 mins and HUVECs were then stimulated with rTp0965 (800 ng/ml) for 24 h. Monocyte THP-1 cells stained with calcein AM were then added in the inserts and the percentage of THP-1 cells in the wells was counted at 4 h under the fluorescence microscope.

To analyze the permeability of HUVEC monolayers after incubation with rTp0965, HRP was added to the transwell inserts, and samples were collected from the wells at designated times. The flux of HRP was calculated. As shown in Fig. 4A, after 1 hour, the HRP permeability in the rTp0965 group was 0.42±0.08, significantly higher than that in control (0.15±0.07, *P*<0.05). After 4 h, the permeability in rTp0965 group was 1.2±0.11, and that in control was 0.52±0.06 (*P*<0.05), suggesting that rTp0965 could induce a remarkable increase in the permeability of HUVEC monolayers.

### Effects of rTp0965 on migration of monocytes across HUVECs

In an *in vitro* static assay of transendothelial migration, we examined whether incubation of HUVECs with rTp0965 would result in an increased migration of monocytes across the monolayer. As shown in Fig. 4 B, addition of rTp0965 (800 ng/ml) to confluent monolayers of HUVECs cultured in transwell inserts resulted in an approximately two-fold increase in the migration of monocyte THP-1 cells at the 4-h time point (35.3% versus 12.7%, *P*<0.001). We next evaluated the effects of Rho signaling on the increase in migration of monocytes across the monolayer induced by rTp0965. We applied the ROCK inhibitor Y-27632 to evaluate the Rho signaling effect. The inhibitor was added to the culture system 30 min before rTp0965 treatment. We found that Y-27632 partially blocked the increase in migration of monocyte THP-1 cells after 24 h of rTp0965 exposure compared with the group treated with rTp0965 but without Y-27632 (19.0% versus 35.3%, *P*<0.05, Fig. 4 B). This observation demonstrated that rTp0965 could up-regulate migration of monocytes across the HUVECs monolayer, and that the ROCK inhibitor Y-27632 could partially block this increase.

### Effects of rTp0965 on F-actin reorganization

**Figure 5:**
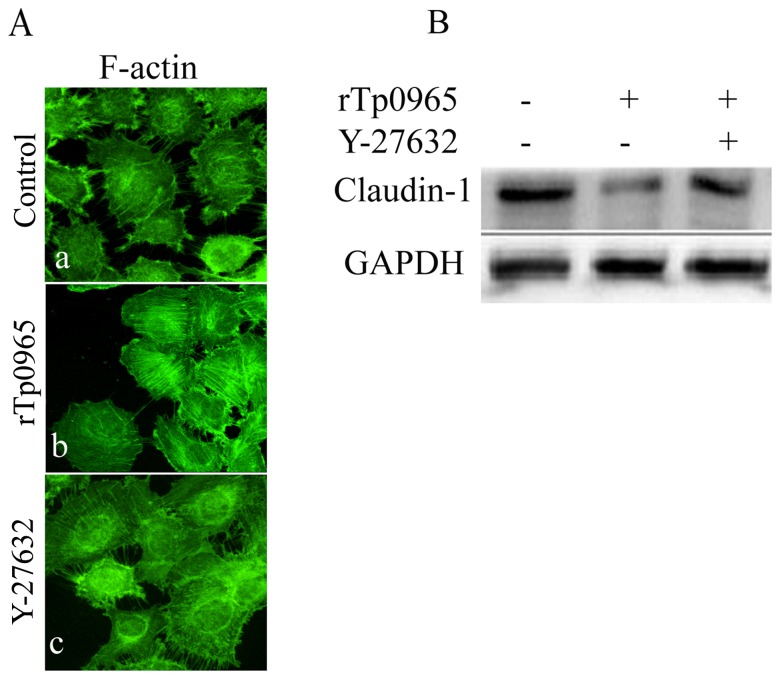
Effects of rTp0965 on F-actin reorganization. Confluent HUVECs were left untreated (a) or were stimulated with rTp0965 (800 ng/ml; b) for 24 h. (**A**). Other HUVECs were preincubated with Y-27632 for 30 mins and then stimulated with rTp0965 (800 ng/ml; c). Cells were then fixed and were stained with rhodamine-phalloidin. (**B**) Effect of rTp0965 on HUVECs expression of claudin-1. Confluent HUVECs were stimulated with rTp0965 (800 ng/ml) or preincubated with Y-27632 for 30 mins and then stimulated with rTp0965 (800 ng/ml) for 2 h. The expression of claudin-1 in total cell lysates were evaluated by Western blot. A representative of three experiments is shown.

To evaluate the effect of rTp0965 on F-actin reorganization in HUVECs, we preincubated HUVECs with rTp0965 and then stained the HUVECs with rhodamine-phalloidin. As showed in Fig. 5 A, in the absence of rTp0965, a rim of F-actin staining was present at the margins of the treated cells, with a few randomly disoriented stress fibers within the cytoplasm. However, in the presence of rTp0965, F-actin rapidly formed organized filamentous networks. These results indicated that rTp0965 could induce F-actin reorganization and distribution in HUVECs.

Previous studies have shown that Rho signaling was involved in the regulation of F-actin induced by LPS in HUVECs. Suspecting a similar regulatory mechanism, we evaluated the effect of Y-27632 on the change induced by rTp0965 on F- actin in HUVECs. We found that Y-27632 partially prevented the F- actin reorganization and redistribution after treatment with rTp0965 (Fig. 5 A), indicating that the Rho signaling was involved in the change of F- actin induced by rTp0965.

### Effects of rTp0965 on HUVECs expression of claudin-1

To assess the effects of rTp0965 on expression of tight junction proteins of HUVECs, we performed Western blot analysis to evaluate changes in level of the tight junction protein, claudin-1. As indicated in Fig. 5 B, after treatment with rTp0965 for 24 h, the level of claudin-1 was markedly decreased in HUVECs and the effect was significantly prevented by the ROCK inhibitor Y-27632. These results indicated that rTp0965 could induce a decline of the claudin-1 level in HUVECs and the ROCK inhibitor could protect against the rTp0965 effect on expression of the tight junction protein.

## Discussion

Previous studies demonstrated that *T. pallidum* and the *T. pallidum* 47 kDa antigen activated cultured HDMECs to up-regulate the expression of adhesion molecules in a concentration and time-dependent manner [20], [21]. In the present study, we examined the effects of rTp0965 on expression of adhesion molecules of HUVECs. The results show that the HUVEC surface expression of ICAM-1 and E-selectin, as well as the mRNA transcription in cytoplasm, are increased after treatment with rTp0965. E-selectin and ICAM-1 are marker molecules of the activated endothelial cells and mediate the adhesion of leukocytes to the vascular endothelium [34]. E-selectin plays a major role in the rolling of circulating leukocytes near the infected site [35]. Following rolling, ICAM-1 mediates the firm adhesion of leukocytes to the activated endothelium leading to subsequent transendothelial migration that is considered necessary for leukocyte migration at the site of inflammation [36], [37].

Activated endothelial cells also release chemotactic cytokines. In this study we investigated the expression and production of the chemokine MCP-1 from HUVECs stimulated with rTp0965. MCP-1 is a member of the C-C chemokine family and is produced and secreted by monocytes, fibroblasts, and vascular endothelial cells [38]. MCP-1 interacts with its CCR2B receptor on monocytes and macrophages to cause chemotaxis [39]. During *T. pallidum* infection, MCP-1 released from endothelial cells can recruit monocytes and macrophages to the site of infection, thereby promoting inflammation. In this study, both mRNA and protein levels of MCP-1 in rTp0965 treated cells were up-regulated and were higher than those of controls at 24 h post-treatment.

From Figs. 2–3, we also found there was no significant difference between time course of mRNA levels and time course of corresponding protein levels. The reason for this phenomenon is as following. As we know, the amount of adhesion molecules on cell surface is regulated by the stored adhesion molecules in cytoplasm and the synthesized adhesion molecules. After induced, the adhesion molecules stored in cytoplasm can be rapidly transferred to the cell surface. However, it may take several hours to synthesize new adhesion molecules. In this study, we speculate that the rTp0965 protein may stimulate the stored adhesion molecules in HUVEC which could rapidly transfer to the cell surface. In addition, we are sorry that we did not determine the half-life of mRNA and corresponding proteins. From Figs. 2–3, the relation between mRNA and protein was obvious, however, the detail mechanism that rTp0965 upregulated the expression of those cytokines may be complex.

We next determined the effects of rTp0965 on the adhesion of THP-1 cells to HUVECs and HUVEC chemoattraction for THP-1 cells. Previously it was demonstrated that the adhesion of leukocytes to endothelial cells was the first step in the process of transendothelial migration [40], [41]. In this study, the adhesion of THP-1 cells to HUVECs was determined in response to rTp0965 stimulus. An increase in the adhesion of THP-1 cells to HUVECs is observed after treating HUVECs with effective stimulatory concentrations of rTp0965 (800 ng/ml). Furthermore, supernatants from HUVECs treated with rTp0965 (800 ng/ml) cause chemotaxis of THP-1 cells. These findings suggested that rTp0965 can stimulate endothelial cells to bind to monocytes and produce certain kinds of chemoattractants for monocytes. Thus, the expression levels of E-selectin and ICAM-1 are sufficient to function as adhesion modules mediating adhesion of monocytes to endothelial cells and the level of MCP-1 is also sufficient to function as a chemoattractant for monocytes.

Transmigration of immune cells is a basic requirement for the protection of tissues by the immune system [42]. Monocytes migration is an important mechanism in the pathogenesis of inflammatory diseases [43], [44], [45]. The present work shows that rTp0965 mediates transendothelial migration of THP-1 cells. The extent of increase in transmigration of THP-1 cells in response to rTp0965 is significantly higher than that for controls. There is an approximately two-fold increase in the migration of monocyte THP-1 cells at the 4-h time point (35.3% versus 12.7%).

Previous studies demonstrate that the endothelium, activated by pathogens or associated proteins, may express cellular adhesion molecules and open its intercellular junctions [46], [47]. The latter contribute to higher permeability of the endothelial barrier, which is followed by transport of macromolecules and transmigration of leukocytes into infection locations [48]. In addition, the transmigration of leukocytes in turn increase the opening of endothelial cells junctions [49]. In this study, permeability assays confirm that rTp0965 induces an increase of HUVECs monolayer's permeability.

Three distinct endothelial zones based on morphological and functional characterization have been described: tight junctions (zona occludens, TJ); adherens junctions (zona adherens, AJ); gap junctions. These zones are composed of numerous transmembrane and cytoplasmic molecules that assemble into complexes and then associate with the cytoskeleton in mature vessels [50], [51], [52]. TJ are composed of multiple transmembrane proteins including JAM1, occludin, and claudins that are envisioned to cluster with certain plaque proteins (ZO-1, ZO-2, MUPP-1), which in turn affiliate with the actin cytoskeleton [53]. Claudins are shown to form dimers that bind to adjacent endothelial cells to form the “seal” of the tight junction [54]. We therefore focused on claudin-1 in this study. Western blot analysis clearly demonstrated that the level of claudin-1 is markedly decreased after HUVECs being exposed to rTp0965 for 24 h. Our data were consistent with those reported in previous studies demonstrating that various stimuli (including protein kinase, proinflammatory cytokines and pathogens) cause down-regulation of claudin-1 in endothelial cells [55], [56], [57].

We next determined the effects of rTp0965 on expression of F-actin reorganization of HUVECs. The data show that rTp0965 induces the assembly of stress fibres and focal contacts in HUVECs. As described in previous studies, the endothelial cells cytoskeleton has a critical role in endothelial permeability [58], [59]. Destabilization of actin microfilaments or disassembly of microtubules results in a hyperpermeable endothelial monolayer. Many normal and pathological extracellular stimuli, such as cytokines, LPS, and virus protein, induce the actin cytoskeleton reorganization (stress fiber formation, F-actin rearrangements) [60], [61], [62], [63]. In response to these stimuli, the RhoA/ROCK signaling pathway plays an important role in regulating actin cytoskeletal organization and dynamics, inducing actin stress fiber and focal adhesion formation [64], [65]. In the presence of Y-27632, the specific inhibitor of ROCK, no significant changes in F-actin are observed. This suggested that rTp0965-induced changes in the actin cytoskeleton also depended on activation of RhoA/ROCK signaling pathway.

ROCK belongs to the AGC (PKA/PKG/PKC) family of serine/threonine kinases and is a major downstream effector of the small GTPase RhoA. Recent studies suggest that the RhoA/ROCK pathway may contribute to the cytoskeleton organization and participate in a wide variety of processes, including adhesion, migration, polarity, cell cycle progression, and differentiation of many cell types [66], [67], [68]. Inhibitors of ROCK increase the endothelial permeability via regulating expression of TJ proteins and actin cytoskeleton organization. Our study shows that a ROCK inhibitor can protect against the effect of rTp0965 on expression of claudin-1 and ZO-1 and the reorganization of F-actin. Moreover, the results also show that the ROCK inhibitor Y-27632 can partially block rTp0965-induced increases of transendothelial migration of THP-1 cells. These results were consistent with previous reports indicating a critical role of Rho in the regulation of TJ protein expression and cytoskeleton organization [69], [70].

In summary, the results of the present study demonstrate that rTp0965 triggers endothelial cell activation by increasing the expression of adhesion molecules and endothelial cell permeability through decreasing expression of TJ protein and alters the actin cytoskeleton organization, which results in monocyte recruitment and migration through endothelial cell layers and results in the dysfunction of the endothelial barrier. Tp0965 protein may play an important role in the immunopathogenesis of late stage syphilis.
